# The Effect of Co-Administration of Death Camas (*Zigadenus* spp.) and Low Larkspur (*Delphinium* spp.) in Cattle

**DOI:** 10.3390/toxins8010021

**Published:** 2016-01-12

**Authors:** Kevin D. Welch, Benedict T. Green, Dale R. Gardner, Clinton A. Stonecipher, James A. Pfister, Daniel Cook

**Affiliations:** United States Department of Agriculture—Agriculture Research Services—Poisonous Plant Research Laboratory, Logan, UT 84341, USA; Ben.Green@ars.usda.gov (B.T.G.); Dale.Gardner@ars.usda.gov (D.R.G.); Clint.Stonecipher@ars.usda.gov (C.A.S.); Jim.Pfister@ars.usda.gov (J.A.P.); Daniel.Cook@ars.usda.gov (D.C.)

**Keywords:** death camas, larkspur, *Zigadenus*, *Delphinium*, methyllycaconitine, zygacine, cattle

## Abstract

In many rangeland settings, there is more than one potential poisonous plant. Two poisonous plants that are often found growing simultaneously in the same location in North American rangelands are death camas (*Zigadenus* spp.) and low larkspur (*Delphinium* spp.). The objective of this study was to determine if co-administration of death camas would exacerbate the toxicity of low larkspur in cattle. Cattle dosed with 2.0 g of death camas/kg BW showed slight frothing and lethargy, whereas cattle dosed with both death camas and low larkspur showed increased clinical signs of poisoning. Although qualitative differences in clinical signs of intoxication in cattle co-treated with death camas and low larkspur were observed, there were not any significant quantitative differences in heart rate or exercise-induced muscle fatigue. Co-treatment with death camas and low larkspur did not affect the serum zygacine kinetics, however, there was a difference in the larkspur alkaloid kinetics in the co-exposure group. Overall, the results from this study suggest that co-exposure to death camas and low larkspur is not significantly more toxic to cattle than exposure to the plants individually. The results from this study increase our knowledge and understanding regarding the acute toxicity of death camas and low larkspur in cattle.

## 1. Introduction

**Figure 1:**
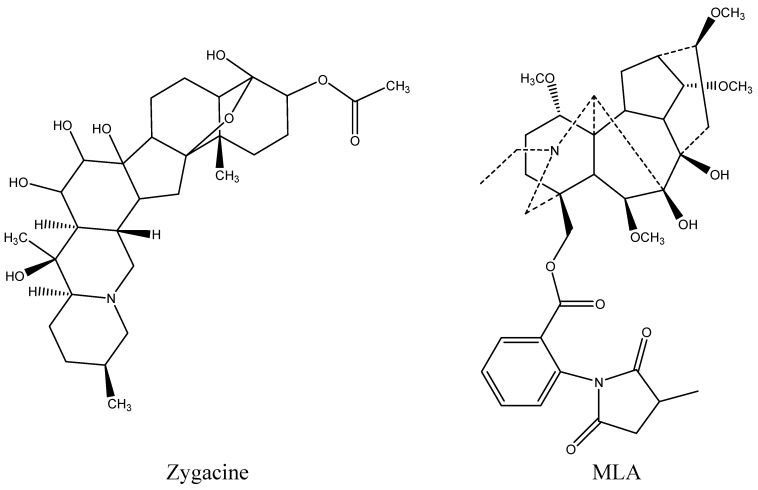
Chemical structure of zygacine, the main alkaloid in *Zigadenus paniculatus*, and methyllycaconitine (MLA), a major toxic alkaloid in the low larkspur *Delphinium andersonii*.

Most rangelands that are used for livestock grazing contain more than one poisonous plant. Two poisonous plants that are often found in the same rangeland are low larkspur (*Delphinium* spp., Ranunculaceae) and death camas (*Zigadenus* spp., Melianthaceae (formerly Liliaceae)). Both of these plants emerge early in the spring and exhibit similar phenological growth stages. Livestock poisonings generally occur during the spring when these plants are abundant and other forage species have little growth. The overgrazing of ranges, wherein higher quality forages have been depleted or management errors result in hungry animals being moved into death camas/larkspur-infested areas, also greatly increases the risk of livestock poisonings [1]. Livestock losses to death camas have been reported in numerous species including sheep and cattle [2,3,4]. The primary effect of death camas intoxication is on the cardiovascular system, often resulting in acute death losses [5]. Zygacine (Figure 1), a steroidal alkaloid, is the primary toxin in death camas, with an LD_50_ of 2.0 mg/kg in mice [6]. Livestock losses to low larkspur also cause large economic losses to cattle producers in the western United States and Canada [7]. The primary result of larkspur intoxication is neuromuscular paralysis, also resulting in death [5]. The toxic compounds in low larkspur are norditerpenoid alkaloids with methyllycaconitine (MLA; Figure 1), an *N*-(methylsuccinimido) anthranoyllycoctonine (MSAL) alkaloid, one of the more prevalent toxins. MLA has an LD_50_ of 4.5 mg/kg in mice [8].

There have been reports of sheep and cattle dying on mountain rangelands wherein both death camas and low larkspur were present (personal communications). However, these specific rangelands did not appear to contain a sufficient density of either plant to have posed a significant risk. Consequently, we hypothesized that co-exposure to death camas and low larkspur could have an additive or synergistic effect, which could lower the toxic threshold for either plant. Recent research has demonstrated that co-treatment of mice with the primary toxins from death camas and low larkspur has an additive effect [6]. However, co-treatment of death camas and low larkspur plants was not any more toxic to sheep than death camas alone [9]. The objective of this study was to determine if the co-administration of death camas would exacerbate the acute toxicity of low larkspur in cattle.

## 2. Results

A dose-response experiment was conducted to determine a dose of death camas, based upon the concentration of zygacine, which would cause minimal signs of intoxication. Holstein steers were dosed at 10, 14, and 18 mg/kg zygacine (Table 1). There was a clear dose-response effect observed. The severity of clinical signs including frothing, lethargy, weakness, dyspnea, and vomiting increased as the dose of zygacine increased. Also, the animals that received larger doses of death camas took longer to recover. Steers dosed with 10 mg/kg zygacine demonstrated no clinical signs of poisoning, and they were able to walk on the treadmill for 5 min at 8 h post-dosing without any signs of weakness. Conversely, steers dosed with 18 mg/kg zygacine demonstrated pronounced signs of poisoning, including frothing, vomiting, and weakness, which was highlighted by the fact that they were not able to walk on the treadmill for 5 min at both 8 and 24 h post-dosing. The steers dosed with 14 mg/kg zygacine did show slight clinical signs of poisoning; however, they were very minor. The steers were able to successfully walk on the treadmill for 5 min at 8 h post dosing.

Death camas is known to cause cardiovascular deficiencies. Consequently, the effects of death camas (DC) and low larkspur (LL) co-treatment on heart rate and EKG were assessed. Four groups of four Holstein steers were compared: alfalfa control (CNT), death camas alone (DC; 2.0 g/kg death camas or 14 mg/kg zygacine), death camas plus low larkspur (DC + LL; 14 mg/kg zygacine and 14 mg/kg MSAL alkaloids), and low larkspur alone (LL; 14 mg/kg MSAL alkaloids). Heart rate in the steers was evaluated for 5 min immediately after a 5 min exercise period on the treadmill at 0, 4, 8, and 24 h post-dosing (Table 2). There was a difference in heart rate between groups (*p =* 0.014). However, there was no difference in heart rate across time (*p =* 0.304) and there was no group × time interaction (*p =* 0.844). All groups that received death camas had a lower heart rate than the CNT and LL groups. However, there was no significant difference between the DC and DC+LL groups (*p =* 0.692). There were no consistent, or obvious, changes in EKG over time or between groups (data not shown).

**Table 1:** Dose-response evaluation of clinical signs of toxicity in Holstein steers fed death camas.

Group	*n*	Plant (g/kg)	Zygacine (mg/kg)	Clinical Signs
10 mg/kg	2	1.4	10.0	No signs
				Able to walk on treadmill for 5 min at 8 h post-dosing
14 mg/kg	2	2.0	14.0	Slight vomiting, lethargy, slight weakness
				Able to walk on treadmill for 5 min at 8 h post-dosing
18 mg/kg	2	2.6	18.0	Frothing, vomiting, significant weakness, dyspnea
				Unable to walk on treadmill for 5 min at 8 and 24 h post-dosing

**Table 2:** The effect of death camas and low larkspur co-treatment on heart rate in Holstein steers.

Treatment	*n*	Death Camas (g/kg)	Low Larkspur (g/kg)	Zygacine (mg/kg)	MSAL (mg/kg)	Heart Rate (BPM)			
						*t* = 0 h	*t* = 4 h	*t* = 8 h	*t* = 24 h
CNT	4	0.0	0.0	0	0	57 ± 4 ^a^	83 ± 2 ^a^	79 ± 25 ^a^	67 ± 13 ^a^
DC	4	2.0	0.0	14	0	53 ± 13 ^a^	55 ± 11 ^b^	53 ± 11 ^a^	65 ± 32 ^a^
LL	4	0.0	4.4	0	14	67 ± 43 ^a^	83 ± 32 ^a^	79 ± 17 ^a^	61 ± 9 ^a^
DC + LL	4	2.0	4.4	14	14	50 ± 9 ^a^	54 ± 13 ^b^	59 ± 12 ^a^	52 ± 6 ^a^

Note: Heart rate was assessed for 5 min immediately after a 5 min exercise period on the treadmill at 0, 4, 8, and 24 h after dosing. Holstein steers were dosed orally with alfalfa (CNT), death camas (DC) alone, death camas and low larkspur (DC + LL), or low larkspur alone (LL). MSAL *= N*-(methylsuccinimido) anthranoyllycoctonine alkaloids. Data represent the mean ± SD of a 4-min selection of the raw trace calculated as beats per minute (BPM) using the cyclic measurements function of the Chart software. A statistical comparison of the heart rate was performed using a two-way ANOVA with a Fisher’s LSD *post hoc* analysis. Within a column, groups that have different superscript letters were significantly different (*p <* 0.05).

The alkaloids in low larkspur act at the neuromuscular junction to inhibit normal muscle function causing severe muscle weakness. The muscle weakness can be exacerbated by physically stressing the animals, *i.e.*, making them walk. Consequently, the effect of low larkspur and death camas co-exposure on exercise-induced muscle weakness and fatigue was evaluated. For this experiment, four groups of four Holstein steers were compared: alfalfa control (CNT), death camas alone (DC; 2.0 g/kg death camas or 14 mg/kg zygacine), death camas plus and low larkspur (DC + LL; 14 mg/kg zygacine and 14 mg/kg MSAL alkaloids), and low larkspur alone (LL; 14 mg/kg MSAL alkaloids). The steers were exercised by walking them on a treadmill at approximately 3 km/h for 5 min at 0, 4, 8, and 24 h post-dosing. The number of steers in each group that were physically able to maintain this pace was noted (Table 3). Statistically, there was no difference (*p* values ranged from 1.0 to 0.14) in the number of steers in each group that were able to walk on the treadmill for 5 min at each time point. However, there was a trend for a greater effect in the steers receiving both death camas and low larkspur, especially at the 4 h time point, where only one of the four DC + LL steers was able to walk on the treadmill for 5 min.

**Figure 2:**
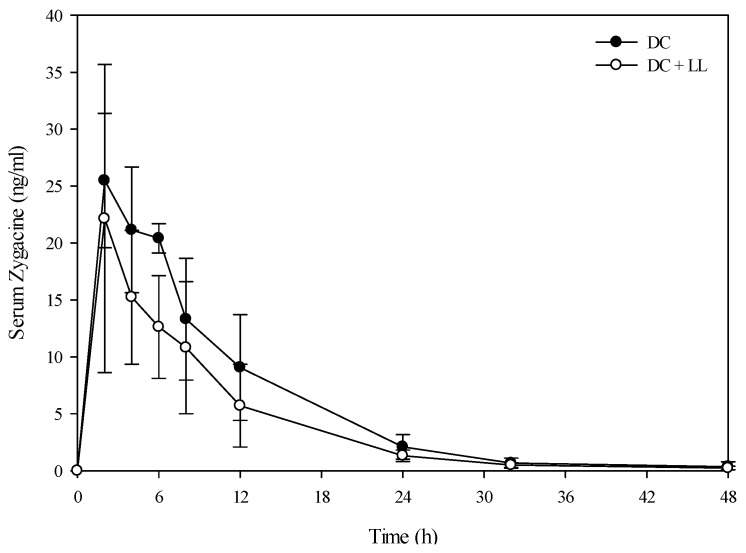
Comparison of the kinetic profile of zygacine in serum from Holstein steers fed death camas alone *versus* death camas and low larkspur. Data represent the serum concentration of zygacine from Holstein steers dosed orally with 2.0 g/kg death camas (DC), which corresponded to 14 mg/kg zygacine. The group that received death camas and low larkspur (DC + LL) was dosed simultaneously with 2.0 g/kg of death camas (14 mg/kg zygacine) and 4.4 g/kg of low larkspur (14 mg/kg MSAL alkaloids). Results represent the mean ± SD of the concentration of zygacine in serum for four steers at each time point. A statistical comparison of the serum alkaloid concentrations was performed using a two-way ANOVA with a Bonferroni *post hoc* analysis. There were no significant differences (*p <* 0.05) observed between the two groups.

**Figure 3:**
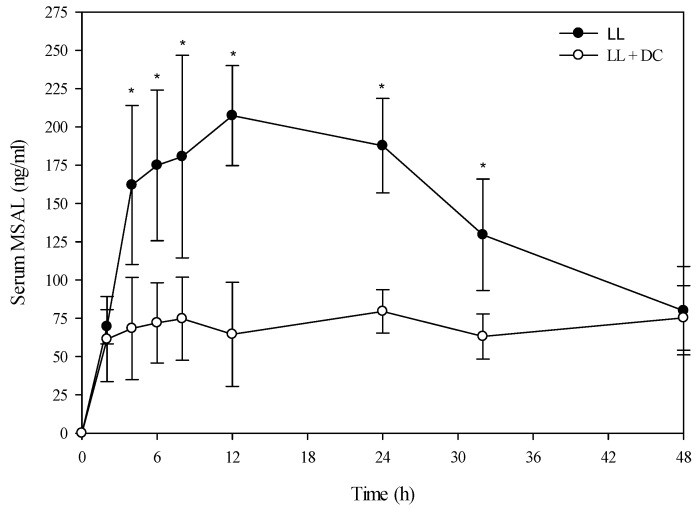
Comparison of the kinetic profile of total MSAL alkaloids in serum from Holstein steers fed low larkspur alone *versus* death camas and low larkspur. Data represent the serum concentration of total MSAL alkaloids from Holstein steers dosed orally with 4.4 g/kg low larkspur (LL), which corresponded to 14 mg/kg MSAL alkaloids. The group that received death camas and low larkspur (DC + LL) was dosed simultaneously with 2.0 g/kg of death camas (14 mg/kg zygacine) and 4.4 g/kg of low larkspur (14 mg/kg MSAL alkaloids). Results represent the mean ± SD of the concentration of total MSAL alkaloids in serum for four steers at each time point. A statistical comparison of the serum alkaloid concentrations was performed using a two-way ANOVA with a Bonferroni *post hoc* analysis. * indicates significant differences (*p <* 0.05) between the two groups.

A toxicokinetic analysis was performed to determine if co-exposure of larkspur and death camas alkaloids altered the kinetic profile of either zygacine or the MSAL alkaloids in cattle. There was no difference (*p =* 0.141) in the serum zygacine concentration between steers dosed with death camas alone *versus* steers dosed with death camas and low larkspur (Figure 2). There was a time effect (*p <* 0.001) with classic first-order kinetics observed, but there was no group × time effect (*p =* 0.682). Additionally, there were no differences in any of the kinetic parameters for zygacine between steers dosed with death camas alone *versus* steers dosed with death camas and low larkspur (Table 4). There was however, a very large difference in the kinetic profiles of the total MSAL alkaloids in the steers dosed with low larkspur alone *versus* steers co-treated with low larkspur and death camas (Figure 3). There were significantly fewer total MSAL alkaloids in the serum of steers dosed with death camas and low larkspur (*p <* 0.001) 4–32 h after dosing. Interestingly, the concentration of serum MSAL alkaloids peaked at 2 h post-dosing and essentially remained constant for 48 h, with no indication of elimination. Consequently, an elimination half-life could not be calculated for the Holstein steers (Table 4). The *T*_max_ was the same between the two groups; however, the *C*_max_, AUC, and absorption half-life all differed between the steers dosed with low larkspur alone and those dosed with low larkspur and death camas (Table 4).

**Table 3:** The effect of death camas and low larkspur co-treatment on exercise-induced muscle weakness in Holstein steers.

Treatment	0 h		4 h		8 h		24 h	
	Yes	No	Yes	No	Yes	No	Yes	No
CNT	4	0	4	0	4	0	4	0
DC	4	0	2	2	3	1	4	0
DC + LL	4	0	1	3	2	2	3	1
LL	4	0	4	0	4	0	4	0

Note: Muscle weakness was assessed by exercising the Holstein steers on a treadmill at 3 km/h for 5 min at 0, 4, 8, and 24 h after dosing. Steers were dosed orally with alfalfa (CNT), death camas (DC), death camas and low larkspur (DC + LL), or low larkspur (LL). Data represent the number of steers that were able to walk for 5 min (yes), or not (no), at each time point. Statistical comparisons were performed using Fisher’s exact test and a Barnard’s test, using a 2 × 2 contingency table; none of the comparisons of the treated groups *versus* the control group were significantly different (*p >* 0.05).

**Table 4:** The effect of death camas and low larkspur co-treatment on zygacine and MSAL alkaloid kinetic parameters.

Breed	Toxin	Group	*T*_max_ (h)		*C*_max_ (ng/mL)		AUC _(0-*t*)_ (ng/mL)		*A*_1/2_ (h)		*E*_1/2_ (h)	
			AVG	SD	AVG	SD	AVG	SD	AVG	SD	AVG	SD
Holstein	Zygacine	DC	2.3	0.5	26	5	283	73	0.7	0.3	5.6	1.3
		DC + LL	1.8	1.2	20	10	196	83	0.5	0.4	5.5	0.7
	MSAL	LL	13.3	0.6	201 *	53	6869 *	1766	5.5 *	2.0	23	13
		DC + LL	15	10	72	25	3154	804	0.9	0.4	N/C	N/C
Beef	Zygacine	DC	1.1	0.6	20	1	185	55	0.2	0.2	5.5	1.4
		DC + LL	1.7	0.4	15	1	161	21	0.4	0.1	6.5	1.5
	MSAL	LL	10.9	4.2	244 *	45	7796	2734	5.4	2.5	12.3	5.2
		DC + LL	6.7	5.2	63	7	3317	424	1.3	1.3	61.0	12.6

Note: The kinetic parameters of absorption half-life (*A*_1/2_), elimination half-life (*E*_1/2_), maximum concentration (*C*_max_), time to maximum concentration (*T*_max_), and area under the curve (AUC_(0-*t*)_) are shown. Statistical comparisons of each kinetic parameter were performed using Student’s *T* test. * indicates a significant difference between groups within a breed. There were no significant differences between breeds. Differences were considered significant at *p <* 0.05. N/C indicates that this kinetic parameter could not be calculated for this group.

**Figure 4:**
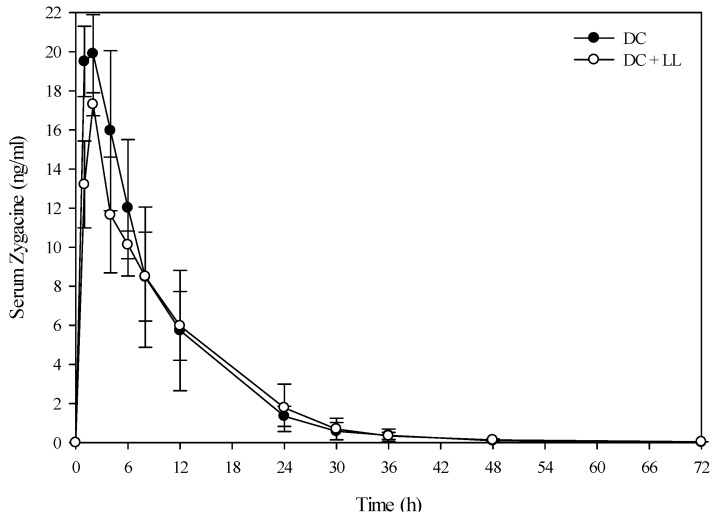
Comparison of the kinetic profile of zygacine in serum from beef steers fed death camas alone *versus* death camas and low larkspur. Data represent the serum concentration of zygacine from beef steers dosed orally with 1.7 g/kg death camas (DC), which corresponded to 12 mg/kg zygacine. The group that received death camas and low larkspur (DC + LL) was dosed simultaneously with 1.7 g/kg of death camas (12 mg/kg zygacine) and 3.7 g/kg of low larkspur (12 mg/kg MSAL alkaloids). Results represent the mean ± SD of the concentration of zygacine in serum for three steers at each time point for the DC group and two steers at each time point for the DC + LL group. A statistical comparison of the serum alkaloid concentrations was performed using a two-way ANOVA with a Bonferroni *post hoc* analysis. There were no significant differences (*p <* 0.05) observed between the two groups.

**Figure 5:**
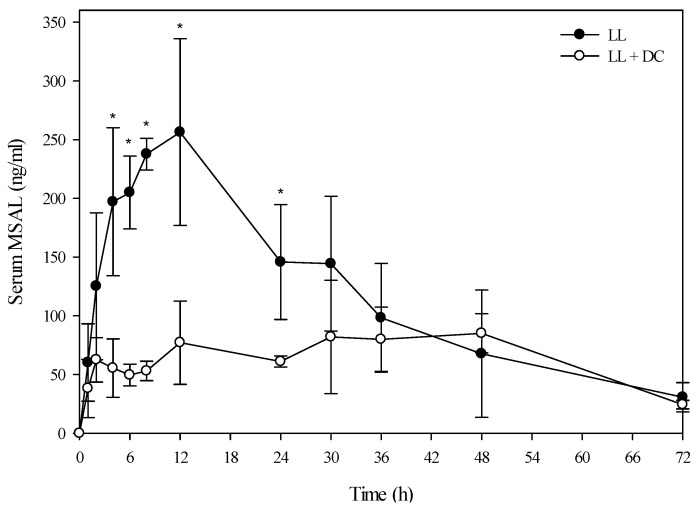
Comparison of the kinetic profile of total MSAL alkaloids in serum from beef steers fed low larkspur alone *versus* death camas and low larkspur. Data represent the serum concentration of total MSAL alkaloids from beef steers dosed orally with 3.7 g/kg low larkspur (LL), which corresponded to 12 mg/kg MSAL alkaloids. The group that received death camas and low larkspur (DC + LL) was dosed simultaneously with 1.7 g/kg of death camas (12 mg/kg zygacine) and 3.7 g/kg of low larkspur (12 mg/kg MSAL alkaloids). Results represent the mean ± SD of the concentration of total MSAL alkaloids in serum for three steers at each time point for the LL group and two steers at each time point for the DC + LL group. A statistical comparison of the serum alkaloid concentrations was performed using a two-way ANOVA with a Bonferroni *post hoc* analysis. * indicates significant differences (*p <* 0.05) between the two groups.

A small group of beef steers (Angus and Hereford) were also dosed with low larkspur alone, death camas alone, and a co-exposure of the two plants to determine if similar results would be observed in beef cattle. The results from the evaluation of the serum alkaloid profiles of zygacine and total MSAL alkaloids were almost exactly the same for the beef steers as the Holstein steers (Figure 4 and Figure 5 and Table 4). The beef steers were also exercised at 0, 4, 8, and 24 h post-dosing by walking them around the corral for 10 min. All of the steers that were dosed with death camas (either death camas alone or death camas plus low larkspur) showed slight signs of poisoning including frothing, vomiting, and slight muscle weakness. However, they were all able to walk for 10 min at each time point (data not shown). There were no noticeable differences in the severity of clinical signs between the beef steers dosed with death camas alone and death camas plus low larkspur.

## 3. Discussion

Most rangelands contain multiple plants that can be poisonous to livestock [2,10]. Consequently, animals are exposed to multiple poisonous plants and thus many different toxins. The basic toxicological information is available for several poisonous plants including the LD_50_, the mechanism of action, and the clinical signs associated with their toxicosis [10,11,12]. However, there is a lack of information available regarding the effects of animals consuming combinations of these poisonous plants. Consequently, it is not known whether consumption of multiple poisonous plants by an animal could have an antagonistic, additive, or synergistic effect. It is possible that the adverse effect elicited by one toxin could potentiate the toxicity of another toxin such that a sub-lethal dose of each toxin, when combined, could produce a lethal result [8,13,14,15]. In this regard, our previous research demonstrated that co-administration of MLA and zygacine to mice had an additive effect [6].

The toxicity of larkspur has been well studied, with the identity and toxicity of the majority of the alkaloids characterized [16,17,18]. The toxic alkaloids in larkspurs are competitive antagonists of nicotinic acetylcholine receptors (nAChR), which results in muscle weakness and death due to respiratory failure [19,20]. Death camas contains steroidal alkaloids, including zygacine, from the same class as those in *Veratrum* species [10,21]. These steroidal alkaloids, including zygacine, are suggested to alter sodium transport in the cardiac nerves [12].

Two previous studies have been conducted to study the effects of co-administration of death camas and low larkspur to animals [6,9]. The results from co-treatment of mice with a 1:1 of zygacine and MLA, suggested that there is an additive effect when combining these two toxins [6]. In sheep, there was not a significant difference in the effect of death camas on sheep dosed with death camas alone *versus* sheep dosed with both death camas and low larkspur [9]. However, sheep are resistant to the toxic effects of larkspur, whereas cattle are very susceptible to larkspur poisoning. Consequently, there is a possibility that in cattle the blockade of nAChRs by larkspur alkaloids could exacerbate the toxicosis caused by death camas alkaloids.

The objective of this study was to determine if the co-administration of death camas would exacerbate the toxicity of low larkspur in cattle. Death camas and low larkspur are commonly found in the same geographical locations in North American rangelands, and growing at the same time [10]. Thus, foraging animals could potentially consume both plants. In this regard, there have been several instances where both sheep and cattle have died, with evidence that the dead livestock had consumed both low larkspur and death camas including the identification of both zygacine and MLA in the blood and gastrointestinal (G.I.) contents from poisoned animals (personal observations). Consequently, it is important to compare the individual and combined effects of death camas and low larkspur in cattle and determine if the co-administration of death camas would exacerbate the toxicity of low larkspur in cattle.

In this study, co-administration of low larkspur and death camas plant material to cattle had a rather minor effect compared to the obvious additive effect observed in mice [6], more similar to the results observed in sheep [9]. There were no large differences or changes in heart rate (Table 2), exercise-induced fatigue (Table 3), or serum zygacine kinetics (Figure 2 and Figure 4) between animals treated with death camas alone *versus* animals treated with both death camas and low larkspur. However, cattle treated with both low larkspur and death camas did appear to have slightly more severe clinical signs of intoxication compared to sheep treated with death camas alone. Although one could argue that this was a subjective observation, there was a trend for cattle dosed with both death camas and low larkspur to show increased exercised-induced muscle fatigue (Table 3).

Interestingly, co-administration of death camas and low larkspur dramatically altered the kinetic profile of the MSAL alkaloids in the serum of both the Holstein and beef steers (Figure 3 and Figure 5). Co-exposure of death camas and low larkspur significantly altered the elimination of the MSAL alkaloids from the serum (Table 4). In fact, there was essentially no elimination of the MSAL alkaloids until 48 h post-dosing (Figure 3 and Figure 5). Conversely, the half-life of elimination of zygacine from blood was approximately 5.5 h, and co-exposure to low larkspur did not affect the zygacine elimination half-life (Table 4). An elimination half-life of 5.5 h indicates that 39 h after maximum concentration is reached (*C*_max_) over 99.2% of the zygacine is eliminated from the blood, which is consistent with the data presented in Figure 2 and Figure 4. The rate of elimination is valuable, as it dictates how long the toxin will remain in the body [22,23]. Therefore these kinetic parameters are useful in evaluating relative risk and to enhance current management recommendations for animals grazing in death camas and low larkspur infested rangelands.

Low larkspur is well known to poison cattle in numerous rangeland settings, and it has been shown to cause numerous large death losses [7]. However, very interestingly, under the conditions used in this study, cattle were not able to be poisoned with a single bolus dose of low larkspur alone to the point that they demonstrated the classic muscle weakness associated with larkspur poisoning. In order to develop muscle weakness to the point that the animals could not remain ambulatory, administration of multiple doses at 13.5 mg/kg MSAL alkaloids (dosed twice daily at 7 am and 3 pm) was required (data not shown). Once the animals received five doses, they demonstrated classical signs of larkspur toxicosis, including an inability to remain ambulatory. This data suggests that the toxicity of low larkspur is somehow different than that of tall larkspur. Numerous studies have demonstrated that when dosing cattle with a single dose of tall larkspur (*D. barberyi* from Manti, UT, USA) at 8 mg/kg, MSAL alkaloids will consistently cause clinical signs of poisoning in most cattle [16,24,25]. One possible explanation for the differences could be due to the different alkaloid composition of tall larkspur *versus* low larkspur. The MSAL alkaloid composition of most tall larkspurs, especially *D. barberyi* from Manti, Utah, the species used in the abovementioned reports, is almost exclusively methyllycaconitine (MLA). However, MLA comprised only about 33% of the MSAL alkaloids in the low larkspur collection used for this study. Thus the dose of the MLA administered to the steers was only 4.7 mg/kg, which is well below the concentration required to cause poisoning in cattle [26,27]. Consequently, if only MLA is “toxic” to cattle then multiple doses of the low larkspur could be required to reach the requisite saturation of nAChRs to cause toxicity. The toxicity of the individual MSAL alkaloids in cattle needs to be addressed in more detail in future studies. Another difference between tall and low larkspur is the presence of non-MSAL type alkaloids in tall larkspurs. Research has demonstrated that even though the non-MSAL alkaloids are less toxic, they can play an important role in the toxicity of tall larkspurs [26,27]. Consequently, it is possible that the low larkspur used for this study was less toxic than a tall larkspur, such as *D. barbeyi*, because it contained less MLA and very little non-MSAL alkaloids.

In summary, the results from this study demonstrate that death camas co-treatment has no significant effect on the toxicity of low larkspur in cattle. Treatment of cattle with death camas caused clear signs of cardiovascular deficiencies including decreased heart rate and muscle fatigue. However, co-treatment with low larkspur did not exacerbate those deficiencies. The results from this study provide an increased knowledge and understanding regarding the acute toxicity of death camas in cattle. For example, the elimination half-life of zygacine from the blood of cattle is approximately 5.5 h, which indicates that after 39 h over 99% of the toxin has been eliminated. Additionally, the results of this study suggest that a toxic, but non-lethal, dose of death camas is 2–2.5 g/kg BW, on a dry weight basis, which would correspond to approximately 5–6 kg of fresh plant material for a 500-kg animal. This information will be useful in further developing livestock management recommendations for ranchers.

## 4. Experimental Section

### 4.1. Plant Material

Death camas (*Zigadenus paniculatus*) was collected in the late pod stage near Logan, UT, USA (N lat 41°46.098′ W long 111°46.688′, at an elevation of approximately 1550 m, PPRL collection 81-9). Low larkspur (*Delphinium andersonii*) was collected in the flowering stage near Picabo, IA, USA (N lat 43°14.813′ W long 114°13.300′, at an elevation of approximately 1475 m, PPRL collection 11-2). Voucher specimens for each plant have been positively identified and deposited in the Poisonous Plant Research Laboratory Herbarium. Each collection of plant material was air-dried and ground to pass through a 2.4 mm screen using a Gehl Mix-All model 55 (Gehl Company, West Bend, WI, USA). After processing, the ground plant material was stored in plastic bags away from direct light at ambient temperature in an enclosed shed until use, with plant alkaloid analyses performed immediately prior to use.

### 4.2. Plant Alkaloid Analyses

Samples of dry ground death camas were extracted following a procedure previously described for larkspur alkaloids [28]. The evaporated extracts were dissolved in 1.0 mL of chloroform and a 0.010 mL aliquot was taken and dissolved in 0.990 mL 50:50 methanol: 0.1% formic acid in an autosampler vial. Analyses were completed using an LCQ Advantage Max (Thermo Scientific, San Jose, CA, USA) mass spectrometer coupled with a Surveyor autosampler plus and MS pump plus (Thermo Scientific) used in-line with a Gemini-NX column (100 × 2 mm; 3 µm; Phenomenex, Torrance, CA, USA) with a guard column of equivalent phase. A binary solvent gradient using 0.1% formic acid (solvent A) and acetonitrile (solvent B), at a flow rate of 0.300 mL/min and the following gradient mixture with time: 10% B (0–1 min); 10%–50% B (1–10 min); 50% B (10–11 min); 50%–10% B (11–12 min); 10% B (12–18 min). The flow from the column was connected to an electrospray ion source. The mass spectrometer was set to a full scan range of 100–800 *m/z* and peak areas were measured from reconstructed ion chromatograms (*m/z* 536.2 selected for zygacine) and were analyzed using purified zygacine (0.5 mg/mL) standard curve at a concentration range of 10, 5, 2.5, 1.25, and 0.625 µg/mL. Injection volume was 5 µL. The death camas used for this study contained 0.7% zygacine (on a dry weight basis).

The low larkspur collection was analyzed for *N*-(methylsuccinimido) anthranoyllycoctonine (MSAL) alkaloid content using HPLC-mass spectrometery, as previously described [29]. The low larkspur used for this study contained 0.2% MSAL alkaloids (on a dry weight basis). The major MSAL alkaloids detected were 16-deacetylgeyerline (16-DAG; 19.50 min), methyllycaconitine (MLA; 20.82 min), geyerline (21.03 min), 14-deacetylnudicauline (14-DAN; approximately 21.25 min), nudicauline (21.91 min), and 14-acetylbearline (24.57 min) [9]. There were also small amounts of non-MSAL alkaloids detected (14.03, 14.91, and 16.72 min) [9].

Alkaloid standards of zygacine and methyllycaconitine were obtained from the Poisonous Plant Research Laboratory’s collection of previously isolated and purified alkaloids. Purity was greater than 95%, as determined by NMR (JOEL, Peabody, MA, USA) and LC-MS analyses. For HPLC-MS analyses acetonitrile was HPLC grade and water was milliQ purified 18.2 MΩ.

### 4.3. Animals

Twenty-two three- to four-month-old Holstein steers and eight five- to six-month-old beef breed steers (Angus and Hereford) weighing 93 ± 14 kg and 133 ± 18 kg, respectively, were used. Animals were maintained on alfalfa for at least one week prior to dosing. Feed was removed from feed bunks the afternoon prior to dosing while retaining *ad libitum* access to water. Plant material was administered in a water slurry via oral gavage at 7 am the morning of the experiment. Two hours after dosing, animals were provided access to alfalfa hay. All procedures were conducted under veterinary supervision and were approved by the Utah State University Institutional Animal Care and Use Committee, #2300.

### 4.4. Death Camas Dose-Response Analysis

Holstein steers were dosed with death camas via oral gavage at 1.4, 2.0, and 2.6 g death camas/kg BW, which corresponded to 10, 14, and 18 mg zygacine/kg BW. Steers were observed for clinical signs of intoxication for 24 h after dosing. A minimum of two cattle were treated at each dose.

### 4.5. Exercise

Holstein steers were trained to walk on a large animal treadmill for one week prior to the start of the study. The steers were dosed orally with dried, ground plant material. Fatigue and weakness were assessed by exercising the cattle 0, 4, 8, and 24 h after dosing. The steers were exercised in groups of two on a treadmill at a rate of approximately 3 km/h for 5 min. If an animal became fatigued to the point that it could not maintain a 3 km/h pace, the treadmill was quickly stopped and the animal was removed. The number of animals that could, and could not, walk for 5 min for each group was noted.

Beef steers were dosed orally with dried, ground plant material. Fatigue and weakness were assessed by exercising the steers 0, 4, 8, and 24 h after dosing. The steers were exercised by walking them in a circle around a 40 m × 10 m corral at a rate of approximately 3 km/h for 10 min. If an animal became fatigued to the point that it could not maintain a 3 km/h pace, it was allowed to fall behind the others. The number of animals that could, and could not, walk for 10 min for each group was noted.

### 4.6. Heart Rate Analyses

Heart rate was monitored in the Holstein steers as outlined previously [16,30]. Heart rate was measured for 5 min immediately after the animals were exercised on the treadmill at 0, 4, 8, and 24 h after dosing. Briefly, data were recorded using an AD Instruments Powerlab, and signals were amplified with an ML132 amplifier (AD Instruments Inc. Colorado Springs, CO, USA). Heart rate was monitored using 3M Red Dot model 2670 repositionable monitoring electrodes (3M Corporation, St. Paul, MN, USA) secured in place with a gel-based formulation of cyanoacrylate adhesive (Henkel Consumer Adhesive, Inc., Avon, OH, USA). The leads were placed as described by Chen *et al.* [31], with the positive electrode placed on the right scapula and the negative electrode on the sternum adjacent to the heart. A ground electrode was attached on the back. The heart rate signal was amplified with a gain range of ±500 µV. The heart rate signal was filtered with a mains filter, 60 Hz notch filter, 120 Hz low-pass; 0.1 Hz high-pass filter, and digital band-pass filter with a high cut-off frequency of 45 Hz and a low cut-off frequency of 0.1 Hz. The cyclic measurements feature of ADI Chart software package was used to calculate heart rate in beats per min (BPM). A 4-min period of heart rate was sampled for calculation of heart rate and reported as BPM.

### 4.7. Serum Analyses

Blood was collected via jugular venipuncture at 0, 2, 4, 6, 8, 12, 24, 32, and 48 h after dosing the Holstein steers. For the beef steers, blood was collected at 0, 1, 2, 4, 6, 8, 12, 24, 30, 36, 48, and 72 h after dosing. The serum fraction of the blood samples was separated by centrifugation (Sorvall, Asheville, NC, USA) at 2800× *g* for 10 min and the serum was stored at −20 °C until analysis.

Matrix matched standards were prepared for methyllycaconitine, a common MSAL alkaloid, and zygacine as follows. A stock solution of methyllycaconitine was prepared at 1 mg/mL in ethanol and then 0.020 mL diluted with 0.980 mL ethanol to provide a 20-ppm solution. A 0.050-mL aliquot was added to 1.950 mL of blank cattle serum and serially diluted with cattle serum to give matrix standards at 500, 250, 125, 62, 31, 16, and 8 ng/mL methyllycaconitine. A 0.020-mL aliquot of zygacine stock solution (0.50 mg/mL in ethanol) was diluted in 0.980 mL of ethanol to give a 10 µg/mL solution, from which 0.020 mL was diluted in 1.980 mL ethanol to give a 100 ng/mL solution. A 0.200-mL aliquot was then diluted with 1.800 mL of blank cattle serum and serially diluted to give zygacine standards at 5, 2.5, 1.25, 0.62, 0.31, and 0.16 ng/mL.

Cattle serum samples were thawed, vortexed, and then centrifuged for 5 min. For the sera samples and the matrix standards, a 0.500-mL aliquot was taken and placed in a 1.5-mL Eppendorf tube. An equal volume of acetonitrile (0.500 mL) containing 250 ng/mL reserpine (Sigma, St. Louis, MO, USA) was added to each sample. Samples were vortexed for 10–15 s and then centrifuged at 16,000× *g* for 10 min. A 0.75 mL aliquot was then removed to a 1.5 mL autosample vial for analysis.

Both methyllycaconitine and zygacine were analyzed by HPLC-tandem mass spectrometry (HPLC-ESI(+)MS/MS). However, because of their different concentrations in the sera they were analyzed under slightly different conditions. For the analysis of methyllycaconitine an LCQ Advantage Max (Thermo Scientific) mass spectrometer coupled with a Surveyor autosampler plus and MS pump plus (Thermo Scientific) was used in-line with a Betasil C18 column (100 × 2.1 mm; 5 µm; Thermo Scientific) with a guard column of equivalent phase. The column was eluted with a binary solvent gradient using 0.1% formic acid (solvent A) and acetonitrile (solvent B), at a flow rate of 0.300 mL/min and the following gradient mixture with time: 15% B (0–1 min); 15%–75% B (1–8 min); 75% B (8–10 min); 75%–15% B (10–11 min); 15% B (11–16 min). The flow from the column was connected to an electrospray ion source. The mass spectrometer was set to scan selected MS/MS experiments during the following time segments: (5.25–6.80 min) MLA parent ion *m/z* 683.3 with CID fragmentation power of 35; and (6.8–10 min) reserpine parent ion *m/z* 609.2 with CID fragmentation power of 33. Reconstructed ion chromatograms used the following selected ions for methyllycaconitine (619.3, 651.3, and 665.3) and for reserpine (397.1 and 448.2). Reserpine was used as an internal standard and calibration and quantitation was completed using peak areas from reconstructed ion chromatograms for methyllycaconitine *versus* reserpine.

For the analysis of zygacine an LTQ Velos Pro (Thermo Fisher Scientific, San Jose, CA, USA) mass spectrometer coupled with a Agilent 1100 binary pump and Agilent autosampler was used in-line with a Betasil C18 column (100 × 2.1 mm; 5 µm; Thermo Scientific) with a guard column of equivalent phase and eluted with a binary solvent gradient using 20 mM ammonium acetate (solvent A) and acetonitrile (solvent B), at a flow rate of 0.300 mL/min and the following gradient mixture with time: 5%–50% B (0–8 min); 50%–100% B (8–14 min) with flow increased to 0.400 mL/min; 100% B (14–18 min); 100%–5% B (18–19 min) with flow reduced to 0.300 mL/min; 5% B (19–25 min). The flow from the column was connected to a heated electrospray ion source. The mass spectrometer was set to scan selected MS/MS experiments during selected time segments: (0–11 min) zygacine parent ion *m/z* 536.2 with CID fragmentation power of 25; and (11–18 min) reserpine parent ion *m/z* 609.2 with CID fragmentation power of 28. Reconstructed ion chromatograms used the following selected ions for zygacine (500.3 and 518.3) and for reserpine (397.1 and 448.2). Reserpine was used as an internal standard and calibration and quantitation were completed using peak areas from reconstructed ion chromatograms for zygacine *versus* reserpine. For HPLC-MS analyses acetonitrile was HPLC grade and water was milliQ purified 18.2 MΩ.

### 4.8. Data Analysis and Statistics

Statistical comparisons of multiple groups were performed using ANOVA with either a Fisher LSD or a Bonferroni *post hoc* test of significance between individual groups using SigmaPlot for Windows (version 12.5, SPSS Inc., Richmond, CA, USA). Statistical comparisons between two groups were performed using a two-tailed, unpaired Student’s *T*-test. The number of cattle that collapsed due to exercise-induced muscle fatigue was analyzed by comparing two groups within a time point using a 2 × 2 contingency table with both a Fisher’s Exact Test and a Barnard’s Test. The alkaloid concentrations were plotted using SigmaPlot for Windows (version 12.5, SPSS Inc., Richmond, CA, USA). Kinetic profiles were analyzed using PKSolver 2.0 [32] using a one-compartment analysis of the data after extravascular input with no lag time. The following parameters were calculated: absorption half-life (*A*_1/2_), elimination half-life (*E*_1/2_), maximum alkaloid concentration (*C*_max_), time to maximum alkaloid concentration (*T*_max_), and area under the curve (AUC_0-*t*_).

## 5. Conclusions

The results from this study demonstrate that death camas co-treatment has no significant effect on the toxicity of low larkspur in cattle. Treatment of cattle with death camas caused clear signs of cardiovascular deficiencies including decreased heart rate and muscle fatigue. However, co-treatment with low larkspur did not exacerbate those deficiencies. The results from this study provide an increased knowledge and understanding regarding the acute toxicity of death camas in cattle. For example, the elimination half-life of zygacine from the blood of cattle is approximately 5.5 h, which indicates that after 39 h over 99% of the toxin has been eliminated. Additionally, the results of this study suggest that a toxic, but non-lethal, dose of death camas is 2–2.5 g/kg BW, on a dry weight basis, which would correspond to approximately 5–6 kg of fresh plant material for a 500-kg animal. This information will be useful in further developing livestock management recommendations for ranchers.
